# “Plug and Play” Photosensitizer–Catalyst Dyads for Water Oxidation

**DOI:** 10.1021/acsami.2c01102

**Published:** 2022-04-28

**Authors:** Ramadan Chalil Oglou, T. Gamze Ulusoy Ghobadi, Ekmel Ozbay, Ferdi Karadas

**Affiliations:** † UNAM—National Nanotechnology Research Center, Bilkent University, 06800 Ankara, Turkey; ‡ NANOTAM—Nanotechnology Research Center, Bilkent University, 06800 Ankara, Turkey; § Department of Electrical and Electronics Engineering, Bilkent University, 06800 Ankara, Turkey; ∥ Department of Physics, Faculty of Science Bilkent University, 06800 Ankara, Turkey; ⊥ Department of Chemistry, Faculty of Science, Bilkent University, 06800 Ankara, Turkey

**Keywords:** organic photosensitizers, photosystem II, PS-WOC dyads, photocatalytic activity, water oxidation catalyst

## Abstract

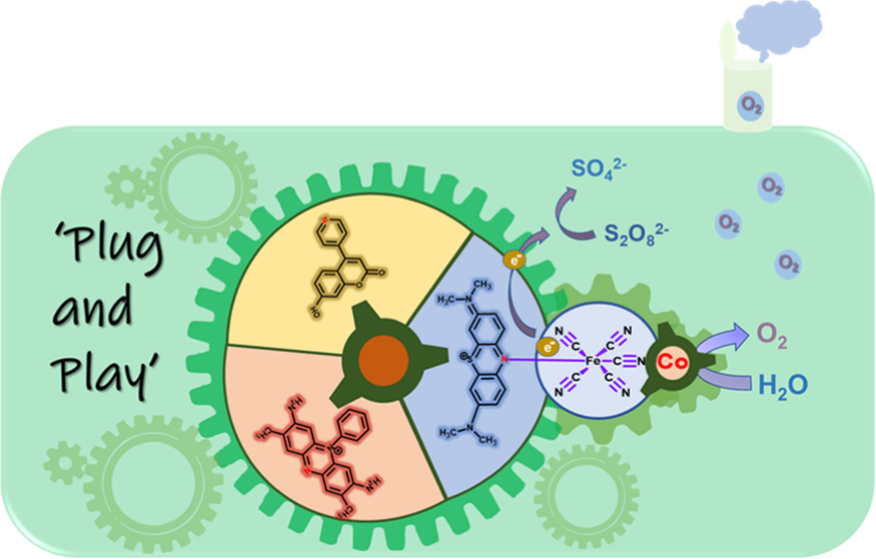

We present a simple and easy-to-scale synthetic method to plug common organic photosensitizers into a cyanide-based network structure for the development of photosensitizer-water oxidation catalyst (PS-WOC) dyad assemblies for the photocatalytic water oxidation process. Three photosensitizers, one of which absorbs red light similar to P680 in photosystem II, were utilized to harvest different regions of the solar spectrum. Photosensitizers are covalently coordinated to CoFe Prussian blue structures to prepare PS-WOC dyads. All dyads exhibit steady water oxidation catalytic activities throughout a 6 h photocatalytic experiment. Our results demonstrate that the covalent coordination between the PS and WOC group not only enhances the photocatalytic activity but also improves the robustness of the organic PS group. The photocatalytic activity of “plug and play” dyads relies on several structural and electronic parameters, including the position of the energy levels of the highest occupied molecular orbital (HOMO) and the lowest unoccupied molecular orbital (LUMO) of the PS with respect to the HOMO level of the catalytic site, the intensity and wavelength of the absorption band of the PS, and the number of catalytic sites.

## Introduction

Building covalently coordinated molecular dyad assemblies has been an attractive approach to mimicking photosystem II, which relies on coupling proper molecular chromophore and catalyst units for efficient charge separation.^1−4^ The selection of the bridging ligand that connects a visible-light-absorbing molecular photosensitizer (PS) to either a hydrogen evolution catalyst (HEC) or a water oxidation catalyst (WOC) plays an essential role in the efficiency of the design. An ideal bridging ligand should have proper functional groups to coordinate the PS and the catalytic unit. Moreover, the studies reveal that the mechanism and the efficiency of charge transfer in donor–bridge–acceptor systems are mainly governed by the size, flexibility, and functional groups in the bridging ligand.^5,6^ For example, a coherent superexchange mechanism could be accelerated by decreasing the length of the ligand, and a transition to a weakly distance-dependent incoherent hopping mechanism could be observed once relatively longer bridging ligands are used.^7−9^ In coherent transport phenomena, conjugated π-systems, such as vinylene and acetylene groups, are preferred for facilitating an electron transfer through the π bonds.^10^ It has also recently been found that the flexibility of the bridging ligand could also affect the charge transfer efficiency.^11^ Therefore, short and rigid bridging groups should be chosen to construct a dyad assembly with an efficient electron transfer. Previously studied PS-WOC dyads are mostly limited to photosensitizer groups such as porphyrin derivatives and ruthenium complexes that absorb light below 465 nm due to the difficulty in designing bridging ligands and properly matching energy levels between the PS and WOC units.^12−17^ Iron-carbene photosensitizers have also recently been utilized for the development of dyads, although to date, only to address photocatalytic H_2_ evolution process.^18,19^

We recently developed a new synthetic pathway to utilize the ability of cyanide bridging group to bind metal ions easily to form a linear M–CN–M′ coordination mode, which provides a facile electronic communication between the metal ions (M–M′ distance is around 5 Å).^20^ The synthesis involves two straightforward steps: First, [Fe(CN)_5_] is coordinated to a pyridyl-containing PS to prepare a molecular Fe(CN)_5_–PS group, [Fe–PS]. Then, the binding ability of the terminal nitrogen atom of the cyanide group to metal ions is utilized to react [Fe–PS] with cobalt ions, which yields a cyanide-based network structure, [CoFe–PS]. We first employed this method to coordinate a ruthenium PS to a CoFe PB structure.^21^ Then, we prepared a [CoFe–porphyrin] compound to develop the first earth-abundant PS-WOC assembly.^22^ We discovered that the porphyrin group serves not only as a light-absorbing component but also as a capping ligand to limit the dimensionality of cyanide-based network structure. The structure of a CoFe–PS architecture mainly differs from the well-known CoFe Prussian blue analogues (PBAs); CoFe–PS could be described as discrete and random-sized cyanide-based CoFe structures surrounded by PS groups, while CoFe PBAs exhibit high crystallinity due to long-range structural order. Furthermore, our controlled experiment with a physical mixture of CoFe PBA and porphyrin reveals that the coordination bond between the porphyrin and [Fe(CN)_5_] groups boosts the photocatalytic water oxidation activity due to enhanced charge transfer between porphyrin and catalytic cobalt sites. We used this method to further develop one of the first earth-abundant dye-sensitized photoanode in the literature.^22,23^ A Prussian blue structure was incorporated into a phenazine-based organic group, Janus green B (JG), on a photoelectrode to prepare a JG-sensitized TiO_2_ photoanode [CoFe-JG]. Irradiation of the working electrode with visible light (λ > 420 nm) under neutral conditions at the potential of 1.23 V_RHE_ exhibits a steady photocurrent density of 50 μA cm^–2^ for over 2 h. In comparison, we prepared a CoFe–Safranine assembly coated on a visible-light-absorbing semiconductor, WO_3_, and we measured photocurrent density of 1.30 mA cm^–2^ at 1.23 V_RHE_ using solar irradiation under mildly acidic conditions (pH 3).^24^

Previous studies point out that cyanide-based heterogeneous PS-WOC assemblies are unique dyad assemblies since the distance between the PS and the catalytic site is minimized, which enhances the coupling between functional units. In one of our previous studies, this design has been highlighted as a “kissing assembly” since a partial mixing of molecular orbitals of PS and WOC units is observed due to short cyanide bridging ligand.^24^ Moreover, the simplicity of the synthesis provides an ideal platform to prepare “plug and play” PS-WOC assemblies with a large agenda of potential photosensitizers. In our pursuit of efficient noble-metal-free bulk devices, we chose a series of low-cost and well-known organic molecules as PS units, which exhibit various band alignments in the photoinduced oxygen evolution process. Each building block, PS, Fe(CN)_5_ group, and Co site, is integrated via a step-by-step strategy to achieve a PS–Fe–WOC coordination mode. This strategy also allows the preparation of PS, [Fe–PS], and [CoFe–PS] compounds separately and the comparison of the optical and structural properties of each component. In this study, we utilized three organic photosensitizers that absorb different regions of the solar spectrum: A green-light-absorbing dye, safranine O ([SF], λ_max_ = 520 nm), is chosen to cover the region with the highest solar irradiation. A red-light-absorbing dye, methylene blue ([MB], λ_max_ = 664 nm), is studied to mimic chlorophyll in Photosystem II, which has not been achieved for a dyad assembly up to date. A UV-light-absorbing coumarin derivative, ([CM], λ_max_ = 334 nm) is studied mainly for comparison. It is also used to elucidate the role of the Co/Fe atomic ratio on the activity since CM is a neutral molecule, while SF and MB are positively charged. In this study, we examined the optical, electrochemical, and photocatalytic properties of PS-WOC dyads to further verify the utility of our approach.

## Experimental Section

### Chemicals and Reagents

All chemicals were used as obtained without any further purification. Cobalt(II) nitrate hexahydrate (Co(NO_3_)_2_·6H_2_O, 99%) and safranin O (C_20_H_19_ClN_4_, >94%) were supplied by Fischer Scientifics. 7-Hydroxy-4-(4-pyridyl)coumarin (C_14_H_9_NO_3_, 95%) was obtained from ABCR. Potassium chloride (KCl, 99–100%), methylene blue (C_16_H_18_ClN_3_S, >97%), and sodium persulfate (Na_2_S_2_O_8_, >98%) were purchased from Sigma-Aldrich. Chloroform (CHCl_3_, >99.9%) was procured by Carlo Erba. Potassium phosphate buffer solution (PBS) was prepared by mixing 0.1 M potassium phosphate monobasic (KH_2_PO_4_, Sigma-Aldrich, 98–100%) and 0.1 M potassium phosphate dibasic (K_2_HPO_4_, Sigma-Aldrich, >99%). Deionized water (resistivity: 18 MΩ·cm^–1^) is used in all experiments. Coumarin, safranin O, and methylene blue photosensitizers are denoted as [CM], [SF], and [MB], respectively, throughout the manuscript. In addition, the iron source of Na_3_[Fe(CN)_5_NH_3_] is denoted as [Fe–NH_3_] and synthesized according to our previously published procedure.^22^

### Synthesis Procedures for [CoFe–CM], [CoFe–SF], and [CoFe–MB]

A two-step synthetic strategy was employed to prepare all PS-WOC assemblies, according to the published procedure.^24^ First, a solution of the organic photosensitizer ([CM], [SF], or [MB]) was prepared with a proper solvent (6 mM [CM] in 100 mL CHCl_3_, 0.03 M [MB] and 0.03 M [SF] in 20 mL H_2_O) and reacted with a solution of Na_3_[Fe(CN)_5_NH_3_] (a 20 mL aqueous solution of [Fe–NH_3_] (0.03 M) for [MB] and [SF] and a 100 mL aqueous solution of [Fe–NH_3_] (6 mM) for [CM]) in a 1:1 stochiometric ratio. The resulting mixture is allowed to stir overnight at room temperature to prepare [Fe–PS] complexes. The suspension is kept in a fridge (+4 °C) overnight to settle down. It is then centrifuged to precipitate [Fe–PS] and decant it. The powder is dried for 2 days in an oven at 75 °C. Next, a solution of [Fe–PS] is reacted with a solution of Co(NO_3_)_2_ (2 equiv). For [CoFe–CM] and [CoFe–MB], [Fe–CM] and [Fe–MB] (0.03 M, 20 mL) were mixed with Co^2+^ (0.06 M, 20 mL) in an aqueous medium, respectively. For [CoFe–SF], [Fe–SF] (0.03 M in 20 mL acetonitrile) is reacted with Co(NO_3_)_2_ (0.06 M in 20 mL ethanol). The solution is stirred overnight at room temperature. The mixture is rinsed several times with cold ethanol and distilled water and then dried in an oven at 75 °C for 2 days to afford the photosensitizer–catalyst dyad assembly, [CoFe–PS]. The bulk precipitates were crushed in a mortar to obtain a powder form for further characterization and photocatalytic experiments.

### Material Characterization

Fourier-transform infrared spectroscopy (FTIR) spectra were measured using a Bruker α Platinum- attenuated total reflection (ATR) spectrometer in the 4000–400 cm^–1^ range with a resolution of 4 cm^–1^ to verify the formation of these complexes. Energy-dispersive X-ray spectroscopy (EDS) analysis and scanning electron microscopy (SEM) were performed by FEI-Quanta 200 FEG to characterize the morphology and depict the atomic ratios, respectively. UV–vis analysis was performed employing an Agilent Technologies Cary 300 UV–vis spectrophotometer. X-ray photoelectron spectroscopy (XPS) (Thermo Fisher Scientific; Al Kα radiation; *h*ν = 1486.6 eV) measurement was also operated at survey mode by operating a flood gun for surface charge neutralization with 30 eV pass energy and 0.1 eV step size, and it was performed for determining the elemental analysis. The correction of peak positions was calibrated by referencing the C 1s peak position (284.8 eV) and shifting other peaks in the spectrum accordingly.

### Photocatalytic Experiments

The amount of photogenerated O_2_ was recorded by gas chromatography (Agilent 7820A GC) equipped with a 5 Å molecular sieve column (Ar as the carrier gas) and a thermal conductivity detector (TCD) detector. The photocatalytic experiments for oxygen evolution were performed in a 10 mL gas-tight Pyrex cell.

The dyad material (10 mg) and Na_2_S_2_O_8_ (20 mM) sacrificial agent were dispersed in a 10 mL of phosphate buffer solution (PBS, pH 7.1). The reaction mixture was degassed with N_2_ gas for 30 min. before each experiment. The reaction flask was coupled to a solar light simulator (Sciencetech, Model SLB-300B, 300 W Xe lamp, AM 1.5 global filter) and calibrated to 1 sun (100 mW cm^–2^). The above solution was also irradiated by visible light illumination (λ > 420 nm) through a 420 nm cut-off filter. The oxygen content in the headspace of the flask was sampled through the septum using a syringe and injected to GC two to three times each time. During the experiments, the mixture was magnetically stirred and no leakage from air in the reaction flask was determined by monitoring the N_2_ content. Each experiment was performed at least three times, and the average of these values is considered as the amount of evolved oxygen. The standard error is calculated by the ratio of the standard deviation of activities for each sampling hour to the square root of the number of samples as shown in the following equation
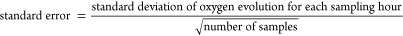


### Electrochemical Experiments

A Gamry Instruments Interface 1000 Potentiostat/Galvanostat was used for electrochemical measurements. FTO substrates with an exposed area of 1 ± 0.05 cm^2^, a Pt mesh counter electrode, and Ag/AgCl (saturated KCl) reference electrode were used in a standard three-electrode electrochemical cell configuration.

The current–voltage (*J*–*V*) curves were measured on electrodes in 0.1 M PBS with a scanning rate of 1000 mV s^–1^ between −0.4 and 1.2 V_Ag/AgCl_ by the cyclic voltammetry (CV) experiments. The potentials are converted to *V*_RHE_ using the Nernst equation
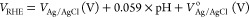
where *V*_RHE_ is the applied potential versus RHE, *V*_Ag/AgCl_ (V) is the applied potential versus Ag/AgCl reference electrode, and *V*_Ag/AgCl_^°^ (V) is the standard potential of the reference electrode (0.197 V_RHE_).

## Results and Discussion

### Characterization Studies

**Scheme 1:**
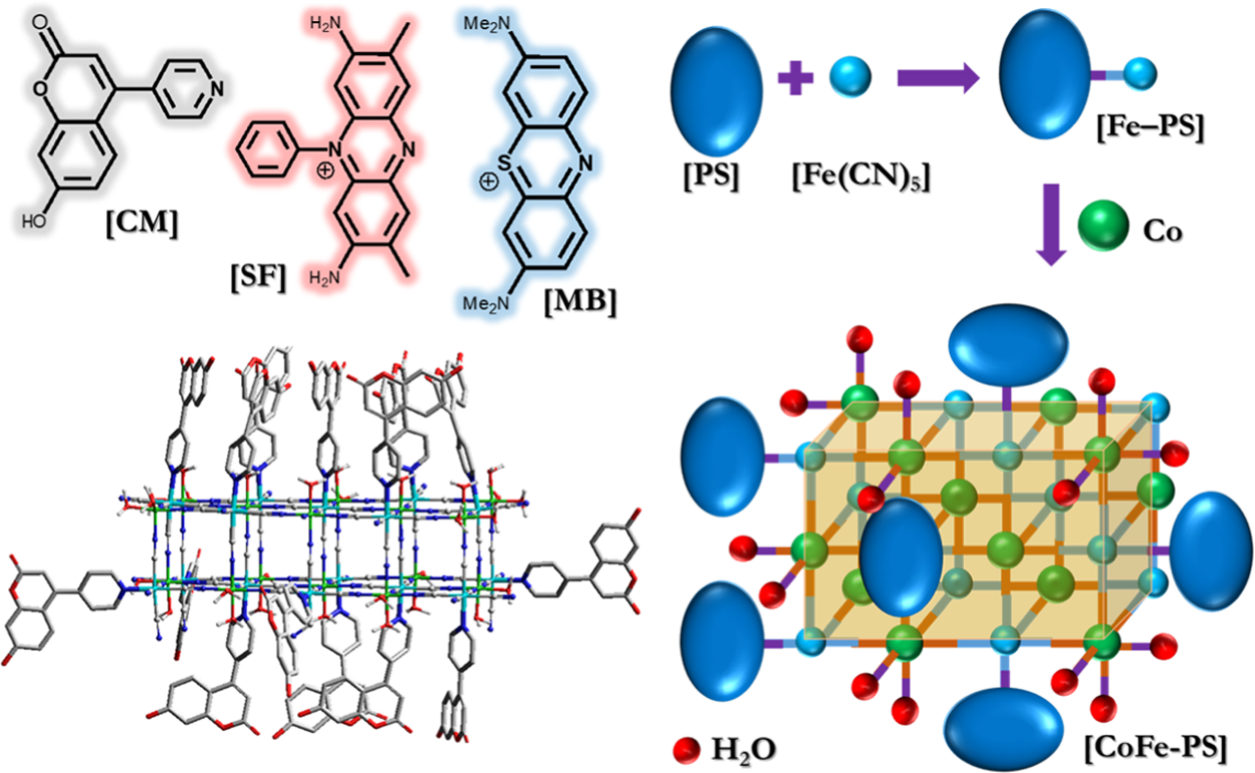
Visual Two-Step Synthetic Construction of PS-WOC Dyad Assemblies The coordination of the PS ([CM], [SF], [MB]) to the [Fe(CN)_5_] group yields molecular [Fe–PS] complexes, which are then reacted with cobalt ions to afford [CoFe–PS] assemblies. [CoFe–PS] exhibits random-sized network CoFe PB structures that are surrounded by PS groups. Catalytic active cobalt sites are surrounded by a combination of N-atom of the CN group and H_2_O molecules.

[CoFe–PS] compounds were prepared via a two-step precipitation method (Scheme 1). First, the NH_3_ ligand in Na_3_Fe(CN)_5_NH_3_ was substituted with a pyridyl-containing PS to produce molecular [Fe–PS] complexes. Then, the lability of the terminal nitrogen atom of the cyanide ligand toward metal ions was utilized to react [Fe–PS] complexes with Co^2+^ ions. The reaction yields the formation of bulk CoFe PB structures, in which the coordination sphere of an iron site is identical to that of [Fe–PS]. On the other hand, cobalt sites are surrounded by a combination of nitrogen atoms of cyanide groups and water molecules, which make them ideal for catalytic water oxidation centers. PS-WOC assemblies with different organic light-absorbing groups are characterized by ATR, SEM, XPS, and UV–vis techniques. [CM], [SF], and [MB] have been examined as PSs for the photocatalytic oxidation of water.

**Figure 1:**
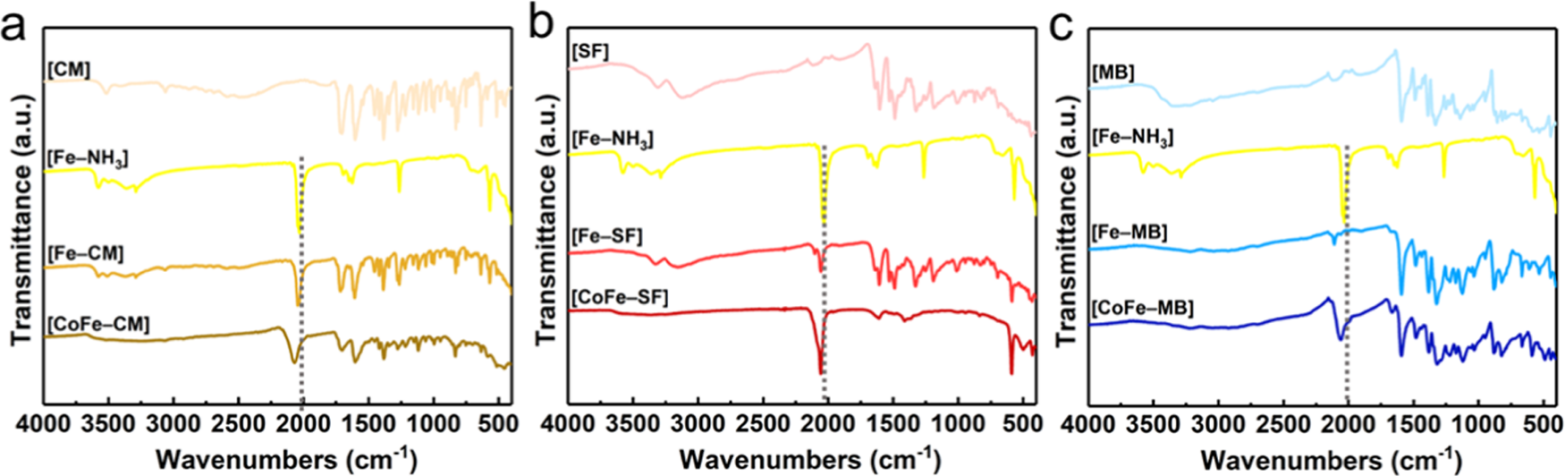
Systematic study of ATR-FTIR spectra for (a) [CM]-, (b) [SF]-, and (c) [MB]-derived assemblies (the perpendicular dash lines demonstrate the shift in the cyanide stretch with respect to [Fe–NH_3_]).

The nature of these organic groups and the coordination mode of cyanide groups in PS, [Fe–PS], and [CoFe–PS] compounds were monitored systematically with Infrared spectroscopy (Figure 1). The organic chromophores exhibit several sharp peaks in the 1600–740 cm^–1^ region, which are assigned to the C–C and C–N ring stretching vibrations.^23^ For [CoFe–CM], one additional peak is observed at 1711 cm^–1^, which is attributed to the carbonyl group.^25^ The revealed peaks at 1610 and 1410 cm^–1^ are decent examples of the presence of α-β unsaturated ketone and C–H of the methyl group for the [CoFe–SF] dyad sample, respectively. For [CoFe–MB], the peaks at 1595 and 900 cm^–1^ are assigned to C=C bands for cyclic alkenes and vinylidene groups, respectively. The cyanide stretch shifts slightly to higher wavenumbers, when [Fe–NH_3_] (νCN = 2040 cm^–1^) reacts with organic PSs to form [Fe–PS] complexes. The CN stretch is obtained at 2040, 2055, and 2107 cm^–1^ for [Fe–CM], [Fe–SF], and [Fe–MB], respectively. The consistent trend in all cases indicates that the PS is coordinated to the iron site and that the electron densities in the iron sites decrease due to the electron-accepting abilities of organic groups.^23,24^ The unusually high shift observed for [Fe–MB] indicates that iron sites are in their Fe^III^ states while [Fe–CM] and [Fe–SF] contain mainly Fe^II^ sites.^26^ νCN shifts further to higher wavenumbers once [Fe–PS] is reacted with Co^2+^ ions, which verifies the Fe–CN–Co coordination mode. νCN stretch is obtained at 2070 and 2057 cm^–1^ for [CoFe–CM] and [CoFe–SF], respectively. Contrary to the [CM] and [SF] cases, a shift to lower wavenumbers is observed for [CoFe–MB] (νCN = 2055 cm^–1^) compared to [Fe–MB] (νCN = 2107 cm^–1^), which could be attributed to the reduction in the oxidation state of Fe sites from 3+ to 2+ with the addition of cobalt ions.^27^ Overall, [CoFe–CM], [CoFe–SF], and [CoFe–MB] depict the characteristic peak of the cyanide group at 2070, 2057, and 2055 cm^–1^, respectively.

SEM analysis is performed to investigate the structure and morphology of [CoFe–PS] compounds. Figure S1 presents randomly evolved bulk particles for [CoFe–CM] while aggregated porous round and plate-shaped structures are observed for [CoFe–SF] and [CoFe–MB], respectively. EDS analysis reveals slightly different Co/Fe atomic ratios. [CoFe–CM], which consists of neutral [CM] groups, exhibits a Co/Fe atomic ratio of 1.43, while positively charged [SF] and [MB] dyes yield lower Co/Fe ratios: 1.17 and 1.22 for [CoFe–SF] and [CoFe–MB], respectively. Based on these analyses, the following empirical formulas are derived: Na_0.14_Co_1.43_[Fe(CN)_5_CM], Co_1.17_[Fe(CN)_5_SF](NO_3_)_0.25_, and Co_1.22_[Fe(CN)_5_MB](NO_3_)_0.17_, for [CoFe–CM], [CoFe–SF], and [CoFe–MB], respectively.

**Figure 2:**
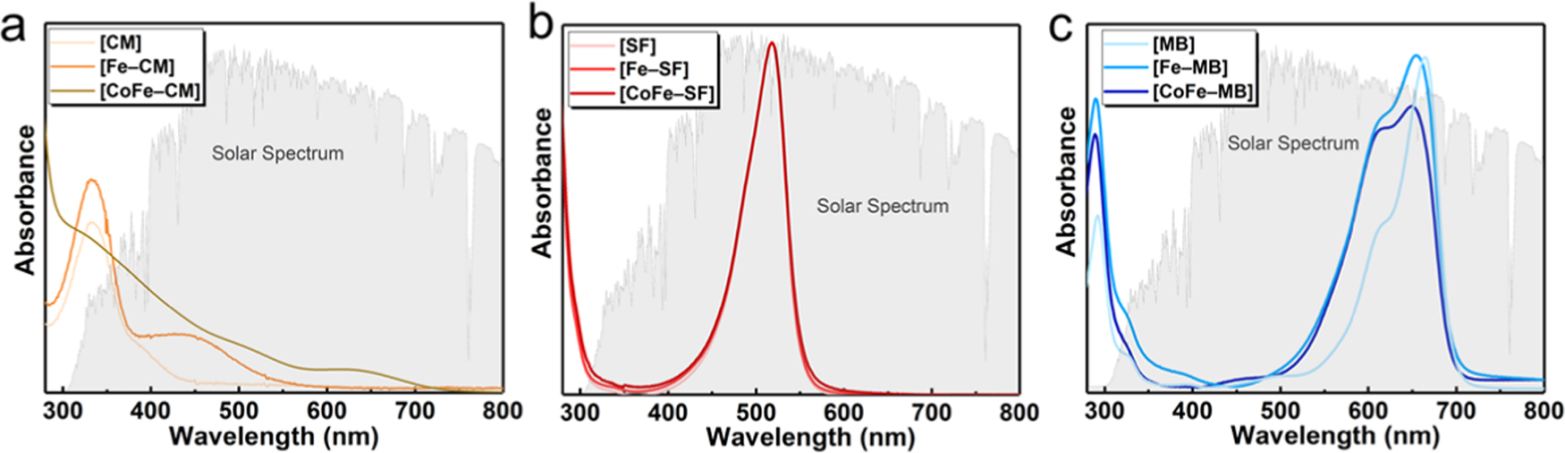
Absorption profiles for 10 μM aqueous solutions of (a) [CM]-, (b) [SF]-, and (c) [MB]-derived compounds. The background depicts the normalized solar spectrum for comparison.

Figure 2 displays the comparison of optical absorption profiles of PS, [Fe–PS], and [CoFe–PS]. Both CM and [Fe–CM] exhibit a dominant absorption mainly in the ultraviolet (UV) region (λ < 400 nm) with a weak molar absorptivity of 2.4 × 10^3^ M^–1^ cm^–1^ (at 334 nm).

When the iron site is coordinated to CM, an additional wide band appears at around 450 nm, attributed to a metal-to-ligand charge transfer (MLCT) transition from the iron site to the CM group, which has been previously observed when [Fe(CN)_5_] group is connected to pyridyl ligands and polymers.^28−30^ The band edge for [Fe–CM] is obtained at 536 nm that corresponds to a band gap of 2.31 eV (Figure 2a).

An additional broad band is observed at around 650 nm due to metal-to-metal charge transfer (MMCT) between Co and Fe sites. All of the bands get broader due to the transition from a molecular structure, [Fe–CM], to a network structure, [CoFe–CM]. On the other hand, the absorption profiles for [MB] and [SF] remain almost similar with the addition of [Fe(CN)_5_] group to form [Fe–PS] and Co ions to afford [CoFe–PS]. MB exhibits a strong absorption band at 664 nm with a shoulder at 617 nm.^31,32^ The intense band at 664 nm shifts slightly to 654 nm for [Fe–MB] and to 650 nm for [CoFe–MB]. The relative intensity of the shoulder at around 615 nm increases gradually from MB to [Fe–MB] and to [CoFe–MB]. This pair of bands also get broader and cover a larger portion of the visible spectrum as MB is coordinated to [Fe(CN)_5_] group. These changes could be attributed to the partial mixing of ligand with metal-based orbitals.^22,24,33^ The absorption bands for MLCT and MMCT processes are not observed for [CoFe–SF] and [CoFe–MB] since (i) they are likely concealed due to the intense absorption bands of [MB] and [SF] in the visible region and (ii) relatively bulky phenazine groups lead to slightly weaker coordination between PS and Fe sites in [CoFe–MB] and [CoFe–SF] compared to [CoFe–CM], which, in turn, afford relatively less efficient MLCT processes. Given the dependence of the MLCT process on the type of the solvent, UV–vis absorption profiles are monitored in dimethylformamide (DMF) as well (Figure S2).^34,35^ The absorption bands that correspond to the π → π* of the organic PS group remain as the major absorption bands for bare PS groups and dyads. In DMF, the MLCT and MMCT bands are more distinguishable for [CoFe–CM] compared to H_2_O. For SF and [CoFe–SF], the π → π* transition that is observed at 518 nm in H_2_O shifts to 535 nm in DMF. More interestingly, a band at 390 nm and a broad one at around 620 nm appear, which could be attributed to the MLCT process from Fe sites to the SF group and MMCT process between metal sites, respectively. Similarly, [CoFe–MB] in DMF reveals an additional broad band at around 510 nm due to the MLCT process. A band due to MMCT is not observed since the strong absorption bands of MB cover most of the 500–700 nm region. [Fe–SF] has a narrow resonant peak in the visible range with an edge positioned at 550 nm resulting in a band gap of 2.25 eV (Figure 2b). [Fe–MB] has a broad visible light absorption profile with an edge at 693 nm and a band gap of 1.78 eV (Figure 2c). Both [Fe–SF] and [Fe–MB] also exhibit weak absorption bands in the UV region. Molar extinction coefficients are found as 5.8 × 10^4^ M^–1^ cm^–1^ at 660 nm for [Fe–MB] and 4.8 × 10^4^ M^–1^ cm^–1^ at 520 nm for [Fe–SF]. Note that their molar absorptivities are comparable to the benchmark [Ru(bpy)_3_]^2+^ photosensitizer (1.46 × 10^4^ M^–1^ cm^–1^ at around 465 nm).^36^ Overall, [Fe–CM] could be described as a UV active molecule, while [Fe–SF] and [Fe–MB] are visible-light-active photosensitizers. Therefore, [CoFe–MB] and [CoFe–SF] display the desired absorption profiles to harvest the visible light portion of the solar spectrum.

### Photocatalytic Water Oxidation Studies

The photoinduced O_2_ evolution performances were evaluated in a neutral 0.1 M PBS containing the powder suspensions of the dyad compound in the presence of a sacrificial electron acceptor under both 1 sun illumination and visible light irradiation (λ > 420 nm). To consume the electrons in the lowest unoccupied molecular orbital (LUMO) level of the PS upon photoexcitation, persulfate anion, S_2_O_8_^2–^, is used as an efficient electron scavenger (ES), which has been commonly used in previous PB-based solar-driven water oxidation studies.^37^

**Figure 3:**
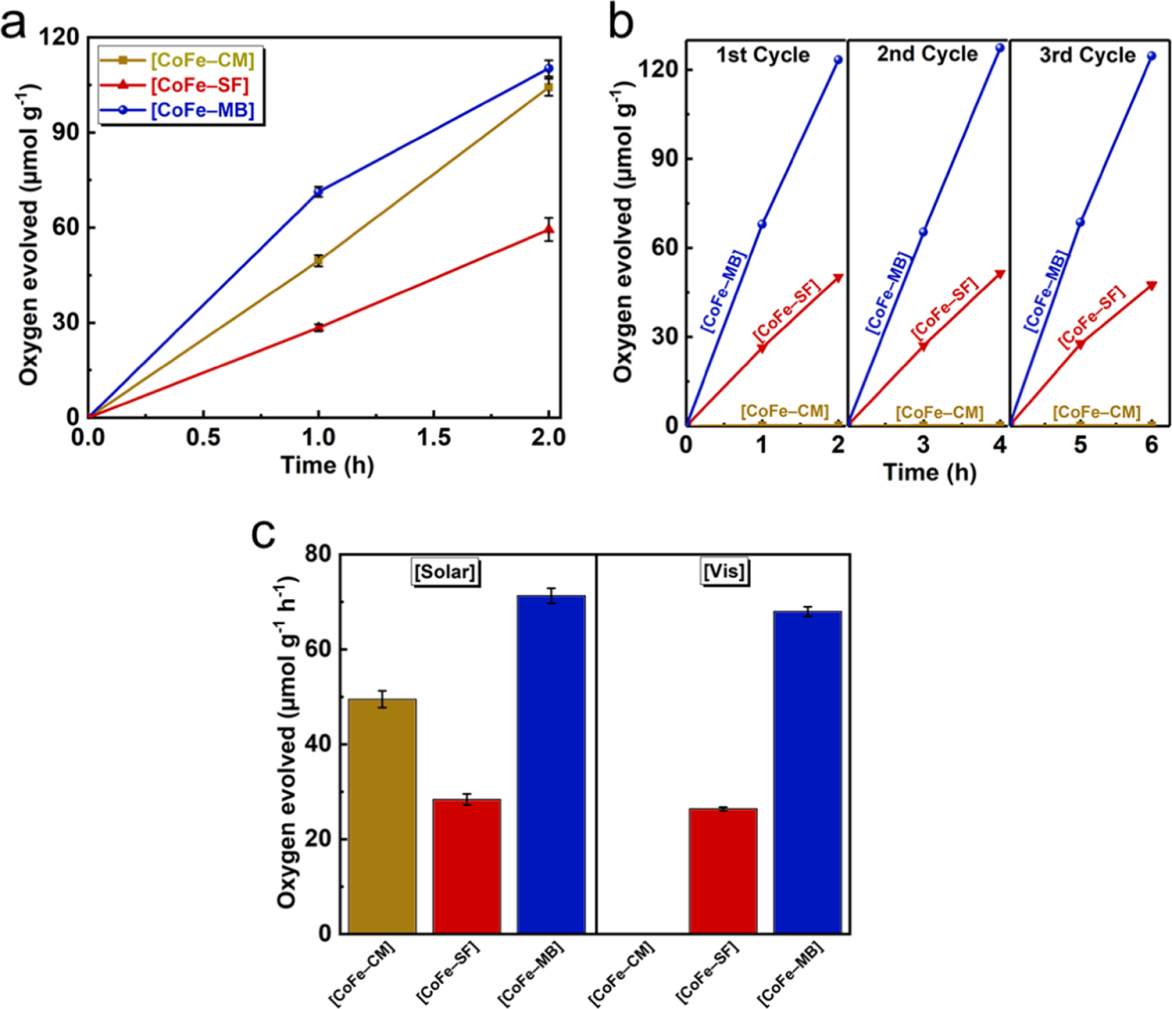
O_2_ evolution rates of [CoFe–CM], [CoFe–SF], and [CoFe–MB]. Experiments are performed with 10 mg of dyad sample and Na_2_S_2_O_8_ (20 mM) at pH 7 in a 0.1 M PBS under (a) solar light irradiation (Xe lamp, 300 W) and (b) visible light irradiation (Xe lamp, 300 W, λ > 420 nm). (c) Comparison of activities based on the first hour of the photocatalytic experiment under solar and visible irradiation.

As shown in Figure 3, a catalytic activity of approx. 71.3 μmol g^–1^ h^–1^ is obtained for [CoFe–MB], which is around 1.4 times higher than that obtained for [CoFe–CM] (49.5 μmol g^–1^ h^–1^) and 2.5 times higher than that of [CoFe–SF] (28.4 μmol g^–1^ h^–1^). Therefore, the following trend is obtained: [CoFe–MB] > [CoFe–SF] > [CoFe–CM] under solar irradiation (Figure 3a). The turnover frequency (TOF) values for the catalysts were also estimated to compare the activity of catalytic active sites. Based on obtained empirical formulas by energy-dispersive X-ray (EDX) analysis, all cobalt sites were assumed to be active sites for the determination of lower-bound TOF values. A similar trend is also obtained for the lower-bound TOF values as the catalytic activities, which are (11 ± 0.26) × 10^–4^ s^–1^, (4.5 ± 0.18) × 10^–4^ s^–1^, and (5.5 ± 0.19) × 10^–4^ s^–1^ for [CoFe–MB], [CoFe–SF], and [CoFe–CM], respectively. Therefore, [CoFe–MB] exhibits a much higher TOF value compared to the previous PB-based photocatalytic water oxidation studies including the ones even with a Ru PS.^21,22^ Since the turnover number (TON) is defined as TOF × time,^38^ which is referred to the number of reactants converted per minute per catalytic site, the following TONs of 3.78 ± 0.09, 1.62 ± 0.07, and 1.98 ± 0.07 are obtained for [CoFe–MB], [CoFe–SF], and [CoFe–CM], respectively. Moreover, [CoFe–SF] and [CoFe–MB] maintain their activities under visible light irradiation (λ > 420 nm) for three consecutive cycles, which is a total of 6 h (Figure 3b). We performed a series of photocatalytic control experiments (Figure S3): (i) No activity is obtained with MB/persulfate and bare persulfate solutions. (ii) A mixture of [CoFe–MB] and persulfate in MeCN does not exhibit any activity, suggesting that the origin of evolved O_2_ is water. (iii) A physical mixture of [MB] and [CoFe] PBA exhibits a much poorer performance with around 4 times lower activity than [CoFe–MB], which proves that the covalent coordination of the PS to the PB structure is essential to boost the charge transfer between the PS and the WOC. Figure 3c displays the O_2_ evolution rates for dyad assemblies under solar and visible irradiation. [CoFe–MB] and [CoFe–SF] exhibit almost the same activities under both solar and visible light irradiation, indicating that their strong absorption bands in the visible region are utilized for light-driven water oxidation process. [CoFe–CM], however, shows no catalytic activity under the same conditions since most of its absorption is located dominantly below 400 nm.

**Figure 4:**
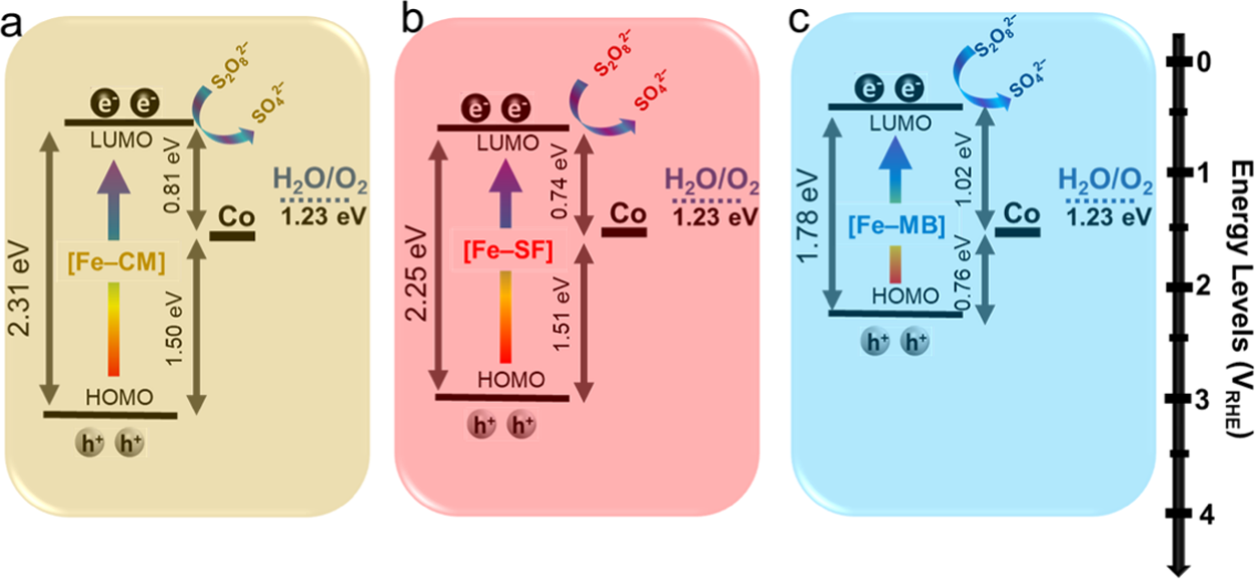
Estimated energy band diagrams for (a) [CoFe–CM], (b) [CoFe–SF], and (c) [CoFe–MB] for the photocatalytic water oxidation process involving the electron transfer mechanism.

A combination of characterization techniques including cyclic voltammetry (CV) and absorption spectroscopy is utilized to estimate the energy levels of [Fe–PS] and Co site with respect to the water oxidation process. All PS-WOC dyads exhibit similar cyclic voltammograms in terms of a redox process attributed to the oxidation of catalytic cobalt sites at around 1.5 V_RHE._^39,40^ Therefore, the oxidation potential for the cobalt site is lower than the water oxidation potential (1.23 V_RHE_), as deduced in our previous studies.^24^ The onset of oxidation and reduction potentials that are extracted from the cyclic voltammogram could be correlated to the LUMO and highest occupied molecular orbital (HOMO) levels of the [Fe–PS], respectively.^41−43^ In our case, the onset reduction potentials (*E*_red_) of [Fe–PS] components are extracted from their CV profiles to assign the LUMO potentials (Figure S4). Optical band gaps are then used to estimate the HOMO levels of [Fe–PS] groups. Based on all of the above-mentioned experimental evidence, the schematic representation of the operation mechanism for light-driven O_2_ production is proposed in Figure 4. The higher photocatalytic activity of [CoFe–MB] compared to [CoFe–SF] and [CoFe–CM] could be justified by their band alignments. The close proximity between the HOMO energy level of [Fe–MB] and Co provides a faster interfacial electron dynamic in [CoFe–MB] compared to [CoFe–SF] and [CoFe–CM]. Furthermore, the LUMO levels of [Fe–SF] and [Fe–CM] are positioned much closer to the energy level of the catalytic Co site compared to their HOMO levels (Figure 4a,b). This mediates electron-hole recombination due to electron transfer from the LUMO level of the PS to the HOMO level of the catalytic cobalt site.^44^ The HOMO and LUMO levels of [Fe–MB] are positioned more properly with respect to the HOMO level Co sites. [Fe–MB] also harvests a larger portion of the solar spectrum due to its strong and broad light absorption that overlaps with the solar spectrum. Therefore, upon excitation with solar light, the holes created in the HOMO level of the [Fe–MB] are transferred to the HOMO level of the Co site to activate it for the water oxidation process and electrons located on the LUMO are consumed with the sacrificial agent, persulfate ions, S_2_O_8_^2–^ (Figure 4c).

In heterogeneous assemblies, the surface concentration of active catalytic sites is also an important parameter that governs catalytic activity.^40,45,46^ The number of active catalytic cobalt sites is estimated by recording Co^2+/3+^ redox wave at different scan rates (Figure S5). All dyad assemblies exhibit a relatively higher surface concentration compared to regular cobalt hexacyanoferrates due to the less crystalline nature of cobalt pentacyanoferrates.^20,47,48^ [CoFe–CM] exhibits a surface concentration of 26 nmol cm^–2^, which is around 25% higher than [CoFe–MB] (21 nmol cm^–2^) and 65% higher than [CoFe–SF] (16 nmol cm^–2^). This trend is also in good accordance with the Co/Fe atomic ratio obtained by EDX analysis. This result indicates that the number of active catalytic sites could also be tuned by the charge of the PS. Relatively fewer cobalt sites are needed to provide a charge balance when positively charged [SF] and [MB] groups are used compared to the neutral [CM] case.

Taking all of the above data into consideration, the origin of the obtained trend in the activity of dyads could be justified as follows: (i) [CoFe–CM] is a UV absorbing PS component, thus under visible light irradiation it has no photocatalytic activity. However, it exhibits a comparable activity under solar irradiation due to its absorption in the UV region and high surface concentration. Both [Fe–SF] and [Fe–MB] could be utilized as visible-light-active photosensitizers. [CoFe–MB] exhibits the highest activity since it exhibits a strong absorption, proper matching of energy levels, and a relatively high number of cobalt sites that can participate in the photocatalytic process.

### Stabilities of Dyads

**Figure 5:**
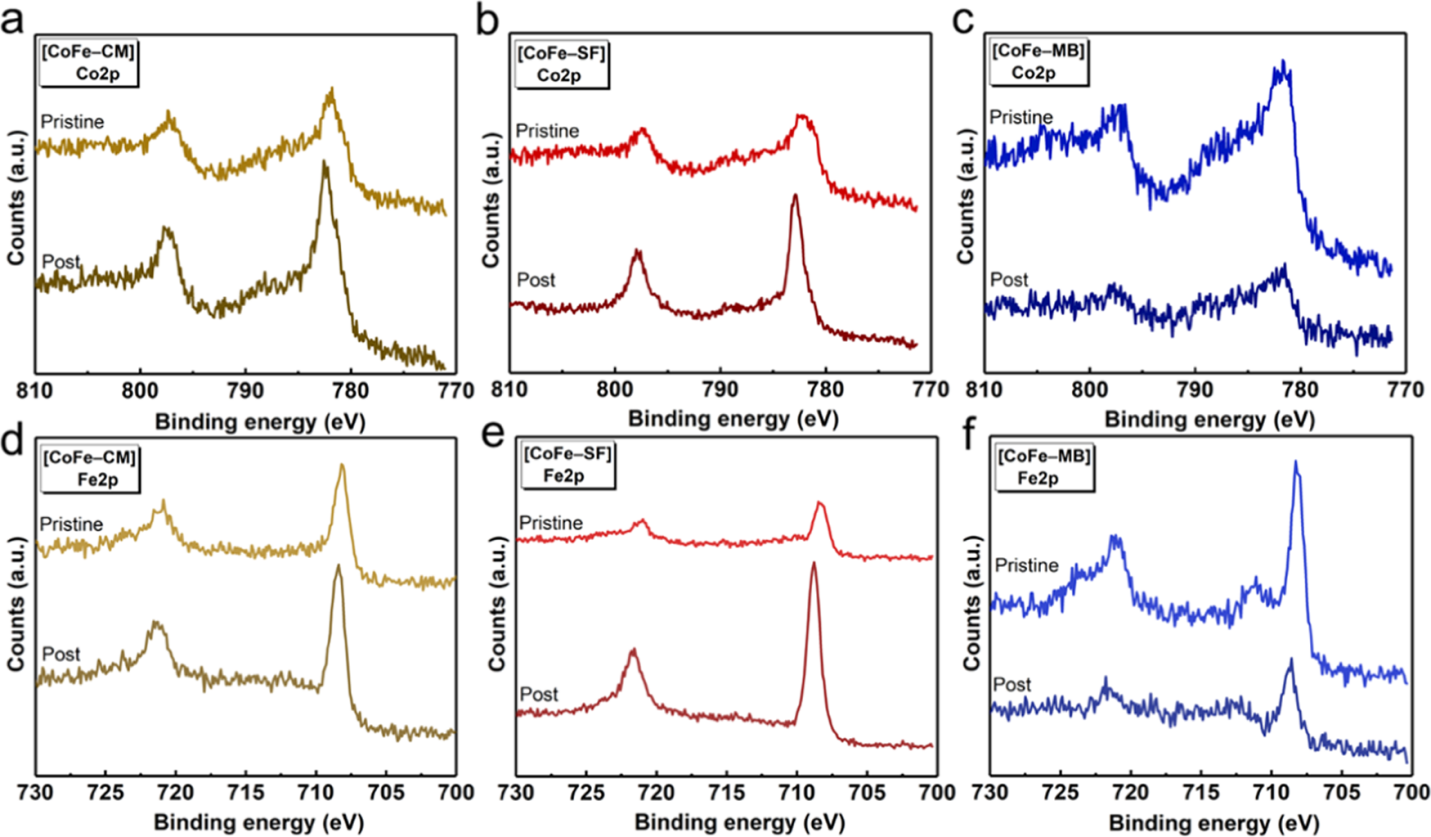
XPS studies performed on pristine and postcatalytic samples: Co 2p for (a) [CoFe–CM], (b) [CoFe–SF], and (c) [CoFe–CM], and Fe 2p for (d) [CoFe–CM], (e) [CoFe–SF], and (f) [CoFe–CM].

Photocatalytic studies indicate that organic PS groups should be covalently coordinated to the CoFe Prussian blue structure for enhanced activity. Since all dyads preserve their photocatalytic activities throughout a 6 h experiment, the decomposition of the PS-WOC dyad via the leaching of catalytic cobalt sites or the degradation of the organic PS group could be ruled out. We also performed characterization studies on the postcatalytic samples to support this thesis. XPS analysis was conducted for both pristine and postcatalytic samples (Figure 5). The Co 2p and Fe 2p signals were conducted in the 811–770 and 740–700 eV regions, respectively. Our previous reports reveal the partial oxidation of cobalt and iron sites after the photocatalytic process.^22,24,49^ Co 2p spectra exhibit two wide bands, one in the 780–785 eV region for Co 2p_3/2_ and another one in the 795–800 eV region due to Co 2p_1/2_. According to the observed Co 2p spectra, a similar trend for the chemical composition (a combination of Co(II) and Co(III) sites) is observed for all dyad assemblies (Figure 5a–c). Both Co 2p_3/2_ and Co 2p_1/2_ peaks exhibit apparent shake-up satellites at the higher binding energy regions due to the presence of Co(II) ions. On the other hand, the Fe 2p region illustrates two apparent peaks for all samples, the one in the 707–712 eV region and the other one at around 721–723 eV corresponding to Fe 2p_3/2_ and Fe 2p_1/2_, respectively (Figure 5d–f). The main observable difference is the secondary peaks for [CoFe–MB] (Figure 5f), which are revealed at 711.15 and 723.64 eV, which suggest a higher concentration of Fe(III) sites in [CoFe–MB] compared to [CoFe–CM] and [CoFe–SF].^50^

XPS studies on postcatalytic samples indicate a slight decrease of the shake-up satellites of Co2p signals of all of the samples and a narrowing in the peaks of [CoFe–CM] and [CoFe–SF] (Figure 5a,b), both of which indicate the partial oxidation of cobalt sites from 2+ to 3+ oxidation states during the photocatalytic process.^51^ Furthermore, the spin–orbit splitting values of Co 2p signals decrease from 15.24 and 15.51 to 15.02 and 15.05 eV for [CoFe–SF] and [CoFe–CM], respectively, which could be attributed to the relative increase in the concentration of Co(III) sites compared to Co(II) sites. However, the spin–orbit splitting of [CoFe–MB] exhibits an increase of 15.48 to 15.97 eV for the postcatalytic sample due to the relative increase in the concentration of Co(II) sites (Note that spin–orbit splitting values of 15 and 16 eV have been reported for [Fe(CN)_6_]^4–^ and [Fe(CN)_6_]^3–^ complexes).^52,53^ In addition, the Fe 2p spectrum of the postcatalytic [CoFe–MB] sample reveals the reduction of the Fe^III^ sites during the water oxidation process as reported in previous studies.^26,27^ The O 1s spectra of all pristine and post samples were examined for a possible metal oxide formation. All O 1s features exhibit binding energies higher than 530 eV, which rule out the possible decomposition of the PB structure to a metal oxide (Figure S6). These results are also in good agreement with the Infrared spectra of postcatalytic samples (Figure S7). The postcatalytic samples of [CoFe–SF] and [CoFe–CM] exhibit a cyanide stretch at 2127 and 2110 cm^–1^, respectively (Figure S7a,b), which are assigned to a Co(III)–NC–Fe(II) coordination mode. For [CoFe–MB]; however, the cyanide stretch is observed at 2087 cm^–1^ (Figure S7c), which corresponds to a Co(II)–NC–Fe(II) coordination mode.^54^ Furthermore, the peaks that correspond to the organic PS group are still present in the postcatalytic samples.

The absorption profiles of [CoFe–SF] and [CoFe–MB] in DMF remain almost similar after the photocatalytic experiment (Figure S8). The intensities of MLCT bands increase slightly with respect to the absorption bands of photosensitizers due to the partial oxidation of metal ions. A significant decrease in the solubility of [CoFe–CM] is observed due to the precipitation of partially oxidized Prussian blue structure surrounded by phosphate counter anions and/or morphological changes during the catalytic process. Nevertheless, a weak absorption band at around 334 nm is observed, which indicates the presence of CM groups in the postcatalytic sample.

## Conclusions

A facile synthetic method was employed to prepare a series of PS-WOC dyad assemblies, which utilizes three photosensitizer groups that absorb different regions of the solar spectrum: a UV-light-absorbing [CM] (334 nm), a green-light-absorbing [SF] (520 nm), and a red-light-absorbing [MB] (664 nm). We found that all three PS-WOC assemblies are active and robust catalytic systems for the photocatalytic water oxidation process. Among these, [CoFe–MB] exhibits the highest activity as high as 68 μmol g^–1^ h^–1^ during a 6 h photocatalytic study under visible light. The relatively high activity of [CoFe–MB] could be attributed to the proper energy level matching between the HOMO and LUMO levels of the PS with respect to the HOMO of catalytic cobalt sites, and relatively high concentration of catalytic cobalt sites. Surprisingly, all organic PS groups including [MB], which degrades easily under photocatalytic conditions, exhibit high stabilities when they are incorporated into the PB network structure. As suggested by our controlled experiments with physical mixtures of PS groups and CoFe PB catalyst, the high activity and stability could be attributed to the efficient charge transfer and separation through short and rigid bridging cyanide groups.

We show that a variety of PSs with different absorption profiles and energy levels, even the ones with a narrow HOMO-LUMO gap and low stability under photocatalytic conditions, could be plugged to cyanide-based PS-WOC dyad assemblies. We demonstrate that this “plug and play” synthetic strategy provides an ideal pathway to employ a large agenda of photosensitizers for photocatalytic applications including dye-sensitized water oxidation. This study offers a guideline for the main parameters that affect photocatalytic activity.

## Supporting Information Available

XPS O 1s spectra, ATR-FTIR spectra, SEM images, photocatalytic OER, and cyclic voltammograms (PDF)
