# Supplementary material for: “Plug and Play” Photosensitizer–Catalyst Dyads for Water Oxidation
Source: ACS Appl Mater Interfaces. 2022 Apr 28;14(18):21131–40. doi: 10.1021/acsami.2c01102 (PMC9100495; doi:10.1021/acsami.2c01102)
Supplement: Supplementary file 1 — am2c01102_si_001.pdf [file am2c01102_si_001.pdf]

SUPPORTING INFORMATION

'Plug and Play' Photosensitizer–Catalyst Dyads for Water Oxidation

Ramadan Chalil Oglou[†], T. Gamze Ulusoy Ghobadi[‡], Ekmel Ozbay[‡,#,¶], Ferdi Karadas[*,†,§]

[†] *UNAM – National Nanotechnology Research Center, Bilkent University, Ankara 06800, Turkey*

[‡]*NANOTAM – Nanotechnology Research Center, Bilkent University, 06800 Ankara, Turkey*

[#] *Department of Electrical and Electronics Engineering, Bilkent University, Ankara 06800, Turkey*

[¶] *Department of Physics, Faculty of Science Bilkent University, 06800 Ankara, Turkey*

[§]*Department of Chemistry, Faculty of Science, Bilkent University, 06800 Ankara, Turkey*

Correspondence

*E-mail: karadas@fen.bilkent.edu.tr

TON and TOF Calculation

TON was obtained by dividing the moles of oxygen evolved from photocatalysis to the moles of cobalt in the catalyst.[1] Since the TOF contains the unit time in its definition, the TON is divided by photocatalysis time in order to calculate the TOF.

$$TON = \frac{the\ moles\ of\ evolved\ O_2}{the\ moles\ of\ Cobalt\ on\ dyad}$$

$$TOF = \frac{TON}{t}$$

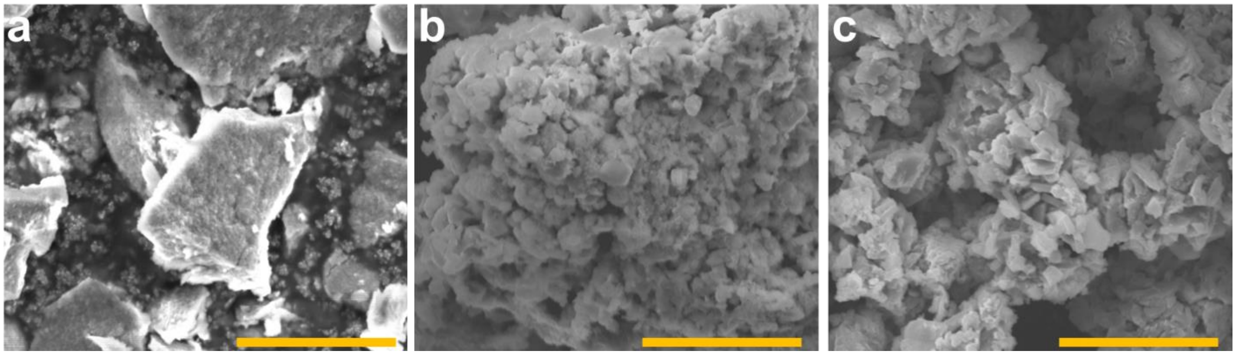

Figure S1. SEM images of (a) [CoFe–CM], (b) [CoFe–SF], and (c) [CoFe–MB] (scale bar: 5μm).

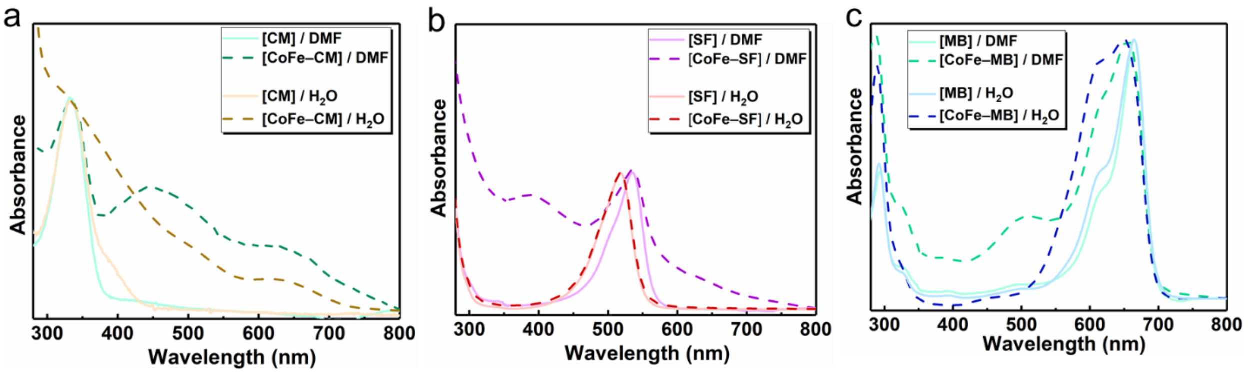

Figure S2. Comparison of absorption profiles for (a) [CM] and [CoFe–CM], (b) [SF] and [CoFe–SF], and (c) [MB] and [CoFe–MB] compounds in aqueous and DMF solutions.

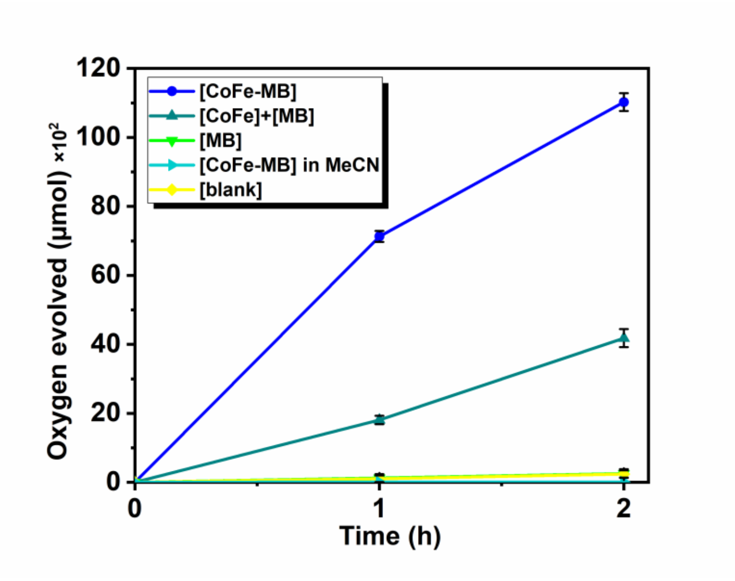

Figure S3. Comparison of photocatalytic performances for [CoFe–MB], physical mixture of [CoFe] + MB, MB alone and blank containing NaS_2O_8 as a sacrificial agent at pH 7 in a 0.1 M PBS under visible light irradiation. In addition, photocatalytic performance of [CoFe–MB] in MeCN electrolyte is demonstrated. All experiments performed with 10 mg of sample.

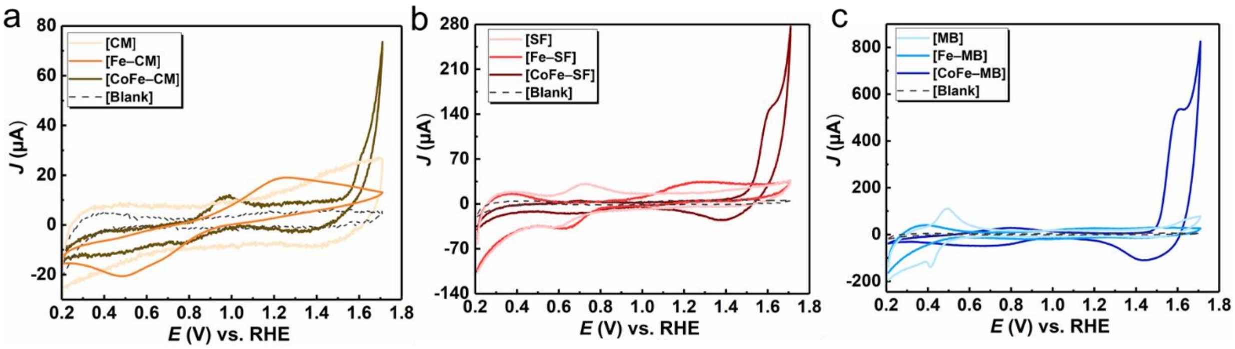

Figure S4. The cyclic voltammograms (CV) of homogeneous solutions (a) CM, (b) SF, and (c) MB based assemblies in 0.1 M PBS (pH 7) at a scan rate of 1000 mV s^{-1}. The CV profile of blank FTO coated glass is represented in dash lines. In each homogeneous measurement, fresh FTO coated glasses were used as a working electrode, Ag/AgCl as a reference electrode, and Pt wire as a counter electrode (($V_{RHE} = V_{Ag/AgCl}(V) + 0.059 \times 7.1 + V^{o}_{Ag/AgCl}(V)$. Assigned reduction potentials for [Fe–CM], [Fe–SF], [Fe–MB] are 0.89 V_{RHE}, 0.77 V_{RHE}, and 0.49 V_{RHE}, respectively. Co oxidations potentials deduced as 1.55 V_{RHE} for [CoFe–CM], and 1.51 V_{RHE} for both [CoFe–MB],and [CoFe–SF].

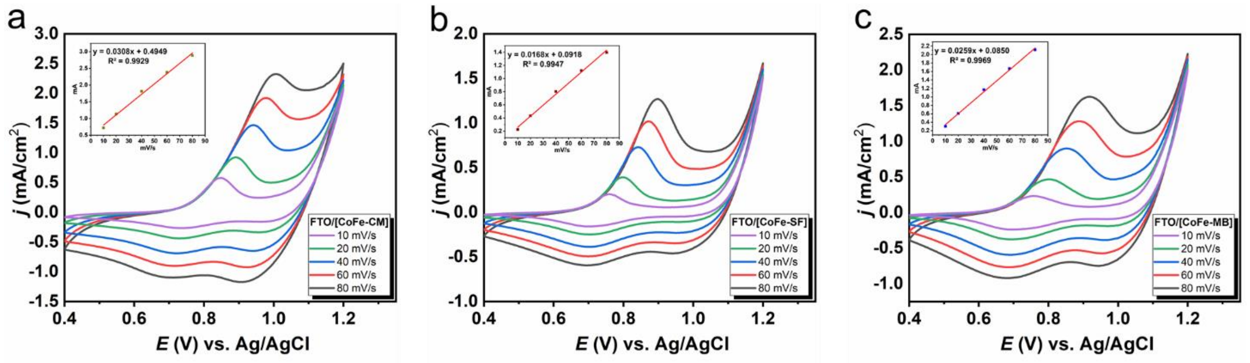

Figure S5. Surface concentrations of active cobalt centers calculated based on Cyclic Voltammograms (CVs) of PS-WOC dyad assemblies which were performed in 0.1 M PBS (pH 7) for (a) FTO/[CoFe–CM], (b) FTO/[CoFe–SF] and (c) FTO/[CoFe–MB] recorded at different sweep rates by using the slope of the linear trend for oxidation currents vs. sweep rates (Inset: The linear relationship between peak current of $Co^{II/III}$ redox couple and sweep rates).[2] The electrodes of PS-WOC systems were prepared by a two-step electrodeposition method.[3,4] First, a well-cleaned FTO electrode was immersed in an aqueous solution of $Co(NO_3)_2$ (10 mM) to afford metallic cobalt particles on the electrode surface by applying a negative potential of –0.9 $V_{Ag/AgCl}$ for 100 s. Then, the rinsed electrode was immersed in an aqueous solution of [Fe–PS] (10 mM) and a chronoamperometric measurement is conducted for 100 s under positive potential of +0.9 $V_{Ag/AgCl}$. Consequentlty, the electrode washed with DI water and kept in oven at 75 ^{o}C for 1 h.

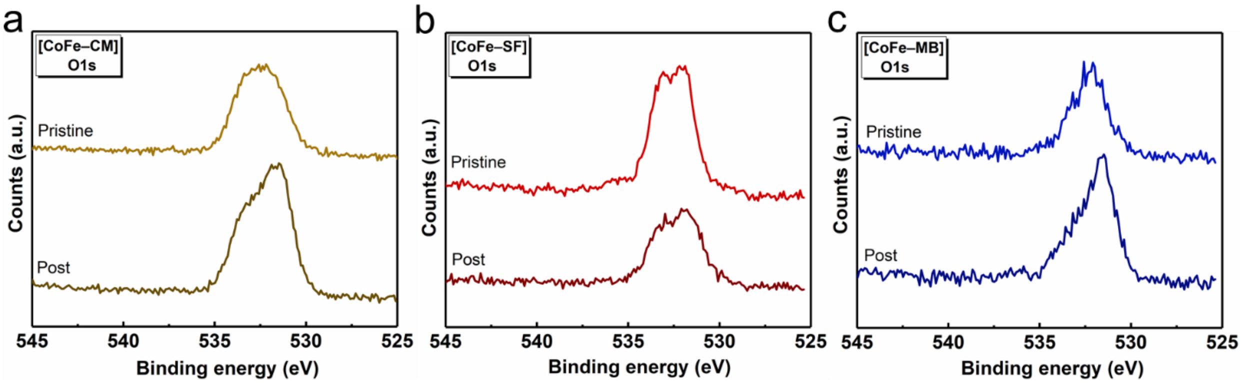

Figure S6. XPS studies of O1s signals for post and pristine (a) [CoFe–CM], (b) [CoFe–SF], and (c) [CoFe–MB] samples.

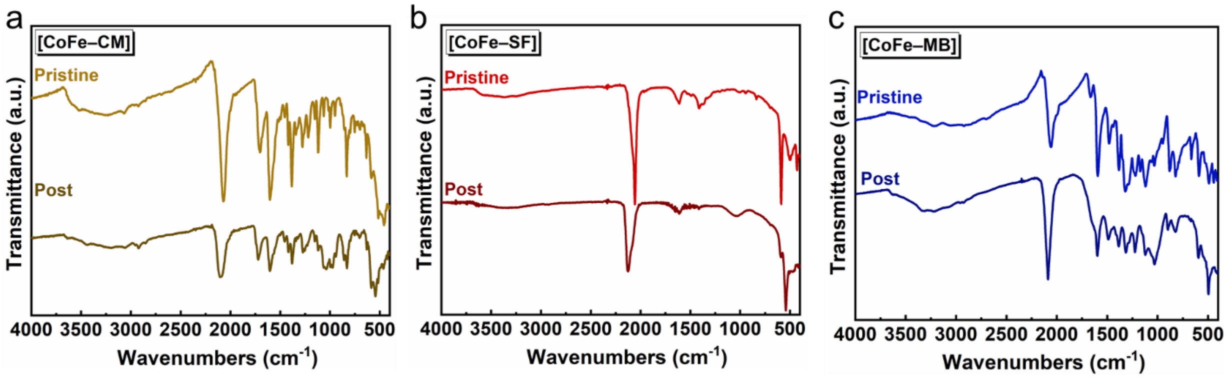

Figure S7. ATR-FTIR spectra for post and pristine (a) [CoFe–CM], (b) [CoFe–SF], and (c) [CoFe–MB] samples. The additional broad peaks that appear in the 1000–1200 cm^{-1} region for post-catalytic samples are attributed to the incorporation phosphate anions into the PB structure as counter anions to provide charge balance in partially oxidized CoFe PB structures.

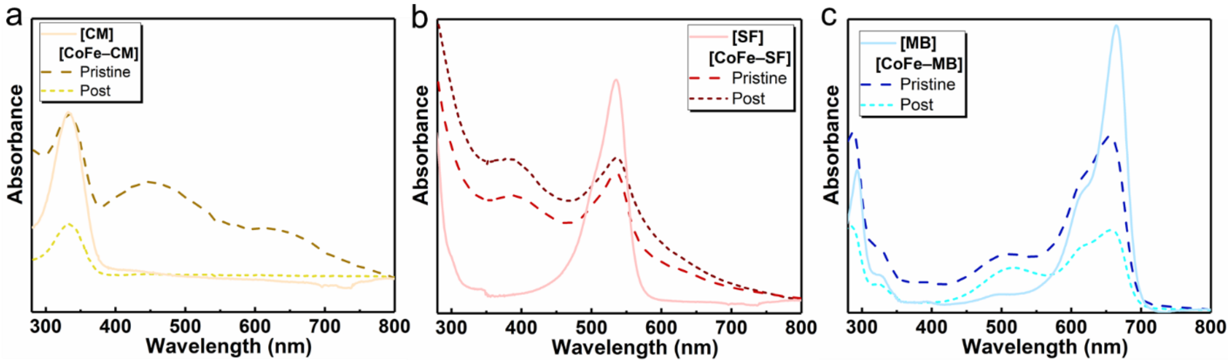

Figure S8. Absorption profiles for DMF solutions of (a) [CM], (b) [SF], and (c) [MB] derived [CoFe–PS] compounds.

REFERENCES

(1) Ahmad, A. A.; Ulusoy Ghobadi, T. G.; Buyuktemiz, M.; Ozbay, E.; Dede, Y.; Karadas, F. Light-Driven Water Oxidation with Ligand-Engineered Prussian Blue Analogues. *Inorg. Chem.* **2022**, *7* (2). https://doi.org/10.1021/acs.inorgchem.1c03531.

(2) Alsaç, E. P.; Ülker, E.; Nune, S. V. K.; Dede, Y.; Karadas, F. Tuning Electronic Properties of Prussian Blue Analogues for Efficient Water Oxidation Electrocatalysis: Experimental and Computational Studies. *Chem. - A Eur. J.* **2018**, *24*, 4856–4863. https://doi.org/10.1002/chem.201704933.

(3) Oglou, R. C.; Ulusoy Ghobadi, T. G.; Ozbay, E.; Karadas, F. Selective Glucose Sensing Under Physiological PH with Flexible and Binder-Free Prussian Blue Coated Carbon Cloth Electrodes. *ChemElectroChem* **2021**. https://doi.org/10.1002/celc.202101355.

(4) Oglou, R. C.; Ulusoy Ghobadi, T. G.; Ozbay, E.; Karadas, F. Electrodeposited Cobalt Hexacyanoferrate Electrode as A Non-Enzymatic Glucose Sensor Under Neutral Conditions. *Anal. Chim. Acta* **2021**, *1188*, 339188. https://doi.org/10.1016/j.aca.2021.339188.
